# Association between venous excess ultrasound score and major adverse kidney events at 30 days in critically ill elderly patients: a multicenter prospective cohort study

**DOI:** 10.3389/fmed.2026.1928971

**Published:** 2026-08-06

**Authors:** Lu Shen, Jiayi Li, Shaohui Fan, Qiancheng Luo, Li Zhong, Weihang Hu, Shijin Gong, Jia Song

**Affiliations:** 1 The Second School of Clinical Medicine, Zhejiang Chinese Medical University, Hangzhou, Zhejiang, China; 2 Department of Critical Care Medicine, Shanghai Pudong New Area Gongli Hospital, Shanghai, China; 3 Department of Critical Care Medicine, The No. 1 People’s Hospital of Huzhou, Huzhou, Zhejiang, China; 4 Department of Critical Care Medicine, Zhejiang Hospital, Hangzhou, Zhejiang, China

**Keywords:** acute kidney injury, elderly patients, intensive care unit, venous congestion, VExUS

## Abstract

**Background:**

Venous congestion is common in critically ill patients and is associated with adverse outcomes, including acute kidney injury (AKI). Elderly patients may be especially susceptible to congestion-related organ dysfunction due to reduced cardiovascular compliance and limited cardiorenal reserve. The venous excess ultrasound (VExUS) score provides a noninvasive bedside assessment of systemic venous congestion. However, its prognostic value in critically ill elderly patients remains unclear. We therefore conducted this multicenter prospective cohort study to evaluate the prevalence of venous congestion assessed by VExUS and its association with adverse kidney events in this population.

**Methods:**

This multicenter prospective cohort study was conducted in the intensive care units (ICUs) of three tertiary hospitals in China. Critically ill patients aged 65 years or older who underwent VExUS assessment within 24 h of ICU admission were eligible for inclusion. Patients were classified into a venous congestion (VExUS ≥2) and a non-congestion (VExUS <2) group. The primary outcome was major adverse kidney events at 30 days (MAKE-30), defined as a composite of serum creatinine reaching ≥200% of the baseline value, initiation of renal replacement therapy (RRT), or death within 30 days of ICU admission. Associations between venous congestion and clinical outcomes were evaluated using logistic regression and Cox proportional hazards regression models. Kaplan–Meier curves were generated for MAKE-30.

**Results:**

One hundred five patients were included in the final analysis, with a median age of 80 years [73–86] and a high prevalence of cardiovascular comorbidities. VExUS ≥2 was observed in 21 patients (20.0%) and was associated with MAKE-30 (adjusted odds ratio [aOR] 5.68, 95% confidence interval [CI] 1.59–27.77; *p* = 0.014). Kaplan–Meier analysis demonstrated a higher cumulative incidence of MAKE-30 among patients with VExUS ≥2 (*p* < 0.001, log-rank test).

**Conclusion:**

Venous congestion is not uncommon within the first 24 h of ICU admission in critically ill elderly patients. VExUS is associated with adverse kidney events in elderly patients and represents a promising bedside tool for the assessment of venous congestion.

**Clinical trial registration:**

https://www.chictr.org.cn/, identifier, ChiCTR2200066987.

## 1 Introduction

Fluid management remains a central yet challenging aspect of care for critically ill patients, especially the elderly (1). Although adequate intravascular volume is necessary to maintain tissue perfusion, excessive fluid administration may result in systemic venous congestion, thereby compromising organ function. Increasing evidence has identified venous congestion as a key contributor to adverse outcomes in critical illness, including acute kidney injury (AKI), prolonged mechanical ventilation, and higher mortality (2, 3). Conventional approaches to assessing venous congestion, such as right heart catheterization, central venous pressure (CVP) measurement, cumulative fluid balance, and physical examination, are limited by invasiveness, poor specificity, or a limited ability to reflect dynamic systemic venous hemodynamics (4–7). Reliable, noninvasive bedside tools capable of providing real-time hemodynamic assessment remain needed.

The venous excess ultrasound (VExUS) score has emerged as a novel and promising method that enables real-time quantification of systemic venous congestion by combining inferior vena cava (IVC) measurement with Doppler ultrasonography of the hepatic, portal, and intrarenal veins (8, 9). Previous studies have shown that elevated VExUS grades are associated with an increased risk of AKI and other adverse outcomes in specific populations, such as patients after cardiac surgery or those with acute coronary syndromes (10–12). However, when applied to general ICU populations, the prognostic performance of VExUS has been inconsistent, with some studies failing to demonstrate an association with clinical endpoints (12, 13). These findings highlight that the prognostic performance of VExUS may vary across different patient populations. Elderly patients, characterized by reduced cardiovascular compliance, impaired venous capacitance, and limited cardiorenal reserve, are particularly susceptible to congestion-related organ injury (14). However, the true prevalence and prognostic significance of systemic venous congestion in this population have not been systematically assessed.

Accordingly, we conducted a multicenter prospective cohort study to investigate the prevalence of venous congestion assessed by VExUS in critically ill elderly patients and to evaluate its association with major adverse kidney events at 30 days (MAKE-30).

## 2 Materials and methods

### 2.1 Study design and setting

This multicenter prospective cohort study was conducted between August 2023 and April 2025 in the intensive care units (ICUs) of three tertiary hospitals in China. The study protocol was approved by an independent ethics committee and was conducted in accordance with the principles of the Declaration of Helsinki. Written informed consent was obtained from each patient or their legal representative prior to enrollment.

Consecutive critically ill patients aged 65 years or older were screened for eligibility. Patients were included if they were expected to remain in the ICU for at least 24 h and underwent a standardized VExUS assessment within 24 h of ICU admission.

Exclusion criteria were as follows, identified based on medical history, echocardiography, or ultrasound findings: (1) atrial fibrillation; (2) major intracardiac shunts; (3) venous outflow obstruction between the right atrium and the assessed venous structures (e.g., Budd–Chiari syndrome, portal vein thrombosis, or hepatic vein thrombosis); (4) history of liver or kidney transplantation; (5) portal hypertension or pulmonary arterial hypertension; (6) end-stage renal disease requiring maintenance dialysis; (7) poor image quality due to morbid obesity (body mass index >40 kg/m^2^) or other technical limitations.

### 2.2 Data collection

All enrolled patients underwent echocardiography and VExUS assessment within 24 h of ICU admission. Baseline demographic and clinical characteristics recorded at enrollment included age, sex, Acute Physiology and Chronic Health Evaluation II (APACHE II) score, Sequential Organ Failure Assessment (SOFA) score, comorbidities, use of diuretics, and administration of vasoactive agents. Laboratory variables measured at the time of ultrasound examination included arterial lactate, serum creatinine, estimated glomerular filtration rate (eGFR), albumin, bilirubin, alanine aminotransferase (ALT), activated partial thromboplastin time (APTT), and brain natriuretic peptide (BNP). During hospitalization, data on ICU length of stay, incidence of AKI and requirement for renal replacement therapy (RRT) were collected. After discharge, patients were followed up by telephone until day 30 to assess vital status and clinical outcomes.

### 2.3 Ultrasound examination and VExUS assessment

Venous congestion was assessed using the VExUS scoring system as originally described by Beaubien-Souligny et al. (8), and all examinations were performed and graded according to the protocol detailed in Supplementary Additional file 1. Bedside ultrasound examinations were performed using portable systems by four physicians who had completed standardized training in point-of-care ultrasound (POCUS) and transthoracic echocardiography prior to study initiation. To ensure measurement consistency, all Doppler recordings were obtained at end-expiration, with simultaneous multilead electrocardiographic monitoring to enable accurate identification of venous waveforms. Images were digitally stored in a standardized format for offline analysis. Final ultrasound images and VExUS grades were independently reviewed by experienced sonographers blinded to all clinical data, with any discrepancies in waveform interpretation or grading resolved by consensus to minimize interpretation bias.

Based on previous literature (8), a VExUS grade of ≥2 was considered indicative of clinically significant venous congestion. Patients were therefore categorized into a venous congestion group (VExUS ≥2) and a non-congestion group (VExUS <2).

### 2.4 Outcomes

The primary outcome was the occurrence of MAKE-30. MAKE-30 was defined as a composite of any of the following events occurring within 30 days after ICU admission: an increase in serum creatinine to ≥200% of baseline; new initiation of RRT; death (15). Baseline serum creatinine was defined as the lowest value in the year preceding ICU admission, reflecting baseline kidney function. In the absence of such historical data, the lowest serum creatinine measured within 24 h after admission was used as the baseline. AKI was defined and staged according to the Kidney Disease: Improving Global Outcomes (KDIGO) guidelines (16).

The secondary outcomes included the prevalence of venous congestion stratified by VExUS grade, as well as 30-day mortality, ICU mortality, and the requirement for RRT during the ICU stay. For patients discharged alive from the ICU within 30 days, follow-up was conducted by telephone on day 30 to assess their clinical status. For those who remained in the ICU at day 30, outcomes were obtained directly from medical records at that time point. For all outcome analyses, the highest VExUS grade recorded on day 1 was used.

### 2.5 Sample size estimation

Based on previous studies, we assumed a prevalence of VExUS ≥2 of approximately 20% and an overall MAKE-30 incidence of around 50%, with an absolute difference of 30% between groups (12, 17). Using a two-sided test for two independent proportions (*α* = 0.05, power = 80%), the required sample size was 115; 105 patients were ultimately enrolled, corresponding to an estimated power of 78%, which is considered acceptable given the anticipated large effect.

### 2.6 Statistical analysis

Continuous variables were expressed as mean ± standard deviation or median (interquartile range), as appropriate, and compared using Welch’s t-test or the Wilcoxon rank-sum test. Categorical variables were expressed as counts (percentages) and compared using the chi-square test or Fisher’s exact test, as appropriate.

Univariable and multivariable regression analyses were used to assess the associations between venous congestion and clinical outcomes. Logistic regression was applied for binary outcomes and reported as odds ratios (ORs) with 95% confidence intervals (CIs), whereas Cox proportional hazards models were used for 30-day mortality and reported as hazard ratios (HRs) with 95% CIs. All multivariable models were adjusted for APACHE II score, SOFA score, and comorbidities, including coronary heart disease (CHD), heart failure, hypertension, and chronic kidney disease (CKD). All prespecified covariates included in the multivariable models were systematically collected and complete for all 105 patients. Kaplan–Meier curves were constructed for MAKE-30 and compared using the log-rank test. All statistical analyses were performed using R software (version 4.0.2), with a two-sided *p* value < 0.05 considered statistically significant.

## 3 Results

### 3.1 Patient characteristics

**Figure 1:**
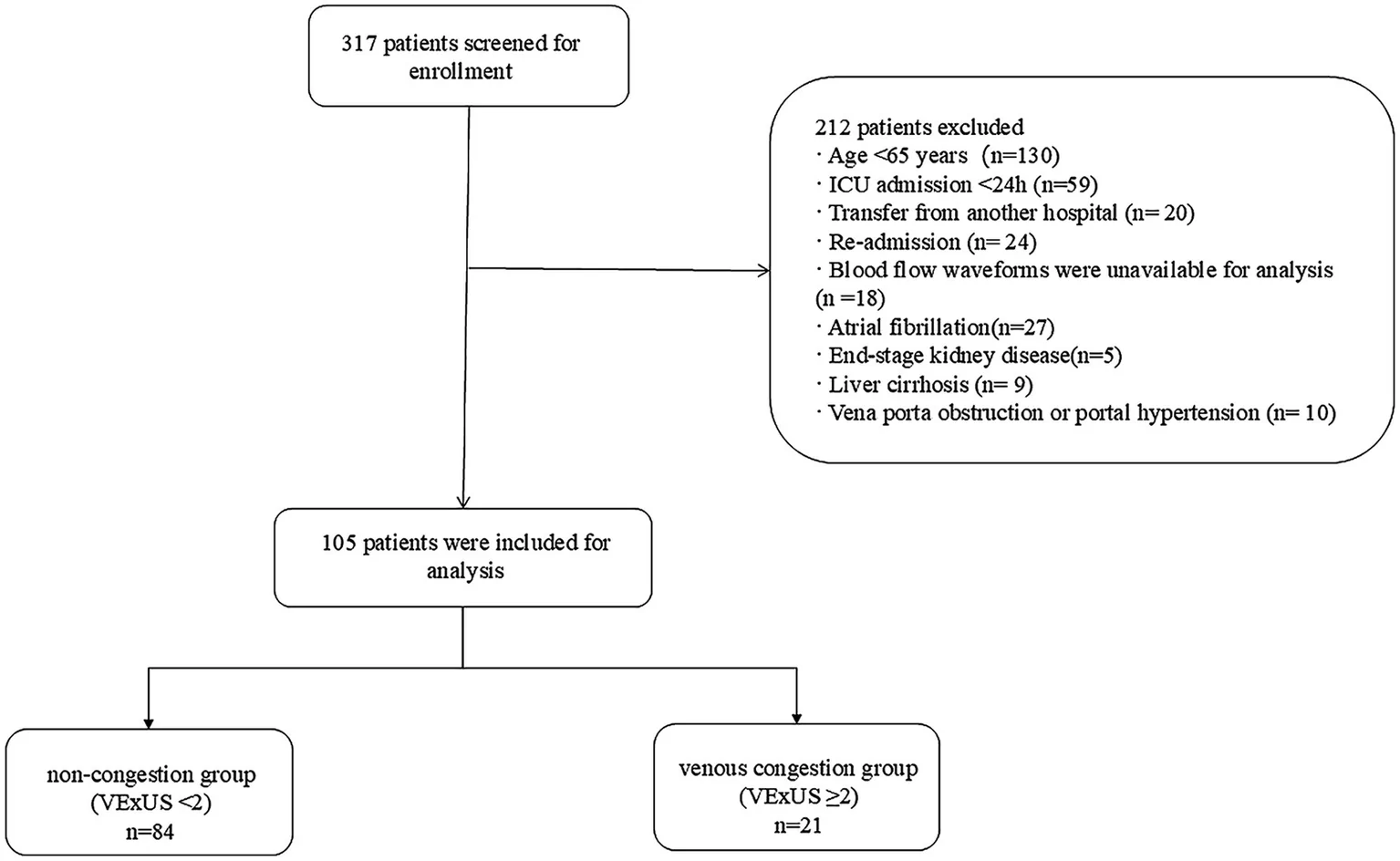
Flowchart of the study. ICU, Intensive care unit; VExUS, Venous excess ultrasound.

During the study period, 317 patients were screened for eligibility. After application of prespecified inclusion and exclusion criteria, 105 critically ill elderly patients were included in the final analysis (Figure 1). The baseline characteristics of the study cohort are summarized in Table 1. The median age was 80 years (73–86), and 72 patients (68.6%) were male. Cardiovascular comorbidities were common, including hypertension (66.7%), CHD (29.5%) and heart failure (27.6%). The mean APACHE II score at ICU admission was 24.1 ± 7.2, and the median SOFA score was 8.0 (6.0–11.0). Sixty-three patients (60.0%) received vasoactive agents, and 39 patients (37.1%) were treated with diuretics. The median ICU length of stay was 13.0 days (6.0–32.0). Within 30 days of ICU admission, 64 patients (61.0%) developed AKI of any KDIGO stage. MAKE-30 occurred in 62 patients (59.0%). Among the individual components of MAKE-30, serum creatinine reached ≥200% of baseline in 50 patients (47.6%), 25 patients (23.8%) initiated RRT, and 29 patients (27.6%) died. These component events were not mutually exclusive.

**Table 1:** Baseline characteristics of patients.

Variable	Overall (*n* = 105)
Demographic characteristics	
Age (years)	80 [73, 86]
Male, *n* (%)	72 (68.6)
APACHE II	24.1 ± 7.2
SOFA score	8.0 [6.0, 11.0]
ICU length of stay (days)	13.0 [6.0, 32.0]
Comorbidities, *n* (%)	
CKD	22 (21.0)
Hypertension	70 (66.7)
Diabetes	35 (33.3)
CHD	31 (29.5)
Heart failure	29 (27.6)
Cerebrovascular disease	32 (30.5)
COPD	12 (11.4)
Cirrhosis	1 (1.0)
Connective tissue disease	3 (2.9)
Malignant tumors	20 (19.0)
Medication, *n* (%)	
Vasoactive agents	63 (60.0)
Norepinephrine	55 (52.4)
Dopamine	6 (5.7)
Epinephrine	3 (2.9)
Vasopressin	2 (1.9)
Metaraminol	9 (8.6)
Diuretics	39 (37.1)
Outcomes, *n* (%)	
ICU mortality	39 (37.1)
MAKE-30	62 (59.0)
30-day mortality	29 (27.6)
AKI	64 (61.0)
Serum creatinine ≥200% of baseline	50 (47.6)
RRT	25 (23.8)

Data are expressed as the mean ± standard deviation, median [interquartile range], or the number of patients (%). APACHE II, Acute Physiology and Chronic Health Evaluation II; SOFA, Sequential Organ Failure Assessment; ICU, intensive care unit; CKD, chronic kidney disease; CHD, coronary heart disease; COPD, chronic obstructive pulmonary disease; AKI, acute kidney injury; RRT, renal replacement therapy; MAKE-30, major adverse kidney events at 30 days. Vasoactive agent categories were not mutually exclusive because some patients received more than one agent.

### 3.2 VExUS grading and group comparisons

Overall, 21 patients (20%) had VExUS grade ≥2 and were classified as having venous congestion. Compared with patients with VExUS grade <2, those with venous congestion had higher levels of BNP (908.0 [291.7–1382.0] vs. 203.0 [107.5–788.0]; *p* = 0.011) and portal vein pulsatility index (PI: 0.6 [0.4–0.6] vs. 0.3 [0.2–0.3]; *p* < 0.001).

**Figure 2:**
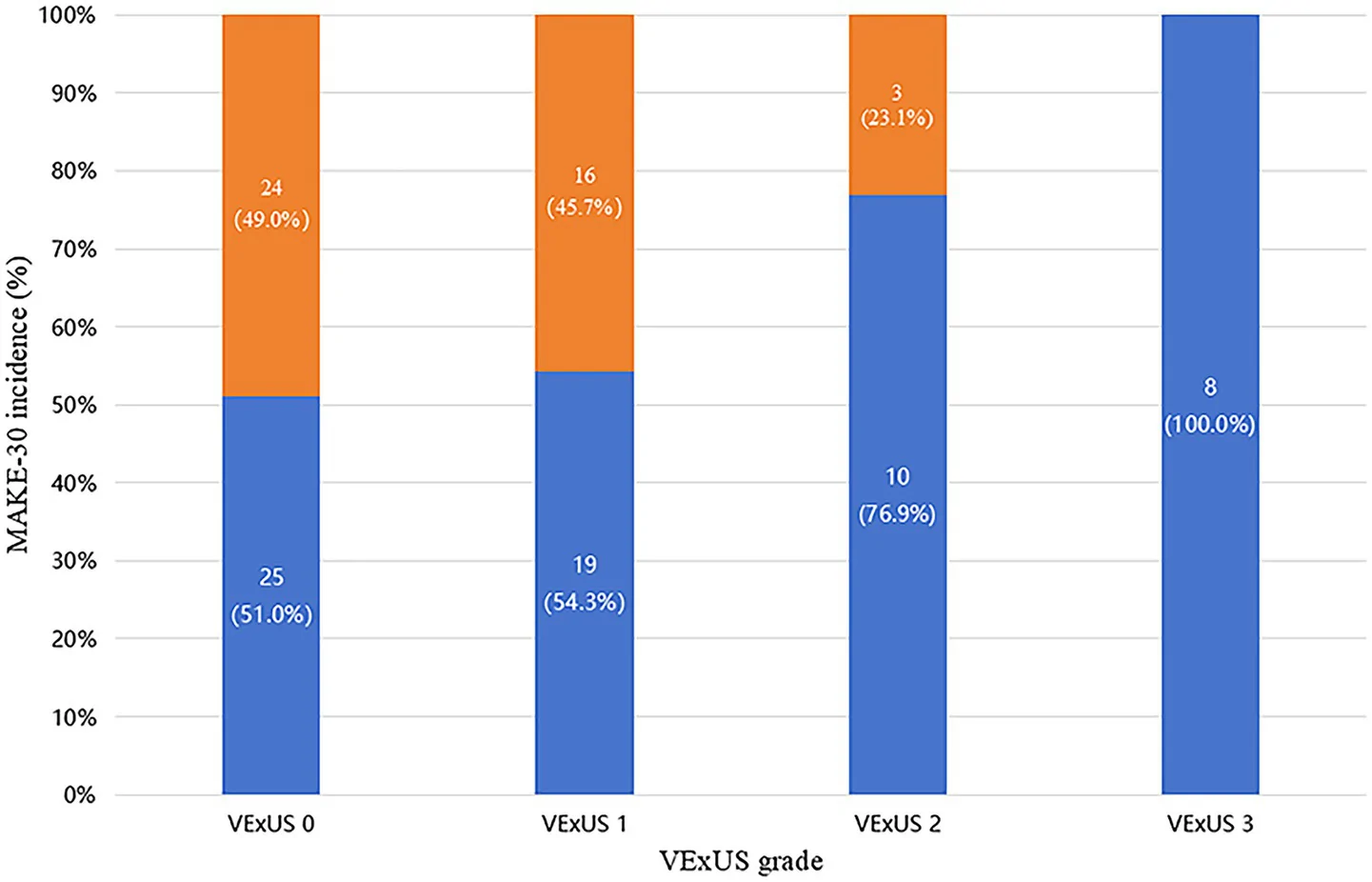
Proportion of MAKE-30 occurrence across different VExUS grades. MAKE-30, Major adverse kidney events at 30 days; VExUS, Venous excess ultrasound.

The incidence of MAKE-30 was significantly higher in patients with VExUS ≥2 than in those with VExUS <2 (85.7% vs. 52.4%, *p* = 0.006). An increase in serum creatinine to ≥200% of baseline was also observed in a higher proportion of patients in the VExUS ≥2 (76.2% vs. 40.5%, *p* = 0.003). No significant differences were observed between the two groups with respect to ICU mortality, 30-day mortality, incidence of AKI, or requirement for renal replacement therapy (Table 2). MAKE-30 incidence increased with higher VExUS grades, with all patients in the VExUS 3 group (n = 8) experiencing the outcome (Figure 2).

**Table 2:** VExUS grading and group comparisons.

Characteristic	VExUS < 2 (*n* = 84)	VExUS ≥ 2 (*n* = 21)	*p*-value
HR (beats/min)	93 [74, 111]	87 [77, 120]	0.863
SBP (mmHg)	123 ± 26	127 ± 22	0.544
DBP (mmHg)	62 ± 15	61 ± 13	0.742
Lactate (mmol/L)	1.8 [1.3, 2.9]	2.5 [1.3, 4.6]	0.255
Creatinine (μmol/L)	97.0 [61.3, 141.9]	134.0 [84.0, 184.0]	0.135
eGFR (mL/min/1.73 m^2^)	57.8 [37.0, 88.2]	37.3 [30.4, 65.3]	0.054
Albumin (g/L)	30.9 [27.9, 34.3]	32.0 [29.0, 35.1]	0.551
Bilirubin (μmol/L)	14.0 [9.9, 20.3]	13.7 [9.8, 24.8]	0.882
ALT (U/L)	24.5 [17.0, 44.5]	21.0 [16.0, 45.0]	0.385
APTT (seconds)	36.1 [30.3, 43.9]	35.3 [30.8, 40.5]	0.674
BNP (pg/mL)	203.0 [107.5, 788.0]	908.0 [291.7, 1,382.0]	0.011
PI	0.3 [0.2, 0.3]	0.6 [0.4, 0.6]	<0.001
IVCMAX (cm)	1.9 ± 0.4	2.2 ± 0.3	<0.001
IVCMIN (cm)	1.4 [1.1, 1.8]	1.9 [1.4, 2.2]	0.011
TAPSE (cm)	1.9 [1.6, 2.2]	1.7 [1.6, 1.9]	0.231
LVOT VTI (cm)	20.7 [15.6, 23.7]	17.8 [13.9, 26.5]	0.832
LVEF, *n* (%)			0.114
<30%	2 (2.4)	2 (9.5)	
30–50%	15 (17.9)	7 (33.3)	
50–70%	59 (70.2)	11 (52.4)	
>70%	8 (9.5)	1 (4.8)	
ICU length of stay (days)	13.0 [6.0, 29.0]	10.0 [7.0, 33.0]	0.807
ICU mortality, *n* (%)	30 (35.7)	9 (42.9)	0.545
MAKE-30, *n* (%)	44 (52.4)	18 (85.7)	0.006
30-day mortality, *n* (%)	23 (27.4)	6 (28.6)	0.913
AKI, *n* (%)	48 (57.1)	16 (76.2)	0.110
Serum creatinine ≥ 200% of baseline, *n* (%)	34 (40.5)	16 (76.2)	0.003
RRT, *n* (%)	17 (20.2)	8 (38.1)	0.086

Data are expressed as the mean ± standard deviation, median [interquartile range], or the number of patients (%). VExUS, venous excess ultrasound; HR, heart rate; SBP, systolic blood pressure; DBP, diastolic blood pressure; eGFR, estimated glomerular filtration rate; ALT, alanine aminotransferase; APTT, activated partial thromboplastin time; BNP, brain natriuretic peptide; PI, portal vein pulsatility index; IVCMAX, maximum inferior vena cava diameter; IVCMIN, minimum inferior vena cava diameter; TAPSE, tricuspid annular plane systolic excursion; LVOT VTI, left ventricular outflow tract velocity–time integral; LVEF, left ventricular ejection fraction; ICU, intensive care unit; MAKE-30, major adverse kidney events at 30 days; AKI, acute kidney injury; RRT, renal replacement therapy. Statistically significant differences between groups (P < 0.05) were observed for BNP and PI.

### 3.3 Association between VExUS ≥ 2 and outcomes

The associations between VExUS ≥2 and clinical outcomes are shown in Table 3. In univariable analysis, VExUS ≥2 was associated with an increased risk of MAKE-30 (OR 5.45, 95% CI 1.69–24.53; *p* = 0.010). After adjustment for APACHE II score, SOFA score, and comorbidities including CHD, heart failure, hypertension, and CKD, this association remained significant (adjusted odds ratio [aOR] 5.68, 95% CI 1.59–27.77; *p* = 0.014). The variance inflation factors were 1.062 for VExUS ≥2, 1.491 for APACHE II, 1.398 for SOFA, 1.126 for CHD, 1.241 for heart failure, 1.015 for hypertension, and 1.096 for CKD. These findings indicate no important multicollinearity among the model covariates, including APACHE II and SOFA. When individual components of MAKE-30 were examined, VExUS ≥2 was independently associated with an increase in serum creatinine to ≥200% of the baseline value (aOR 4.25, 95% CI 1.38–15.05; *p* = 0.016). No statistically conclusive association was detected between VExUS ≥2 and 30-day mortality (adjusted hazard ratio [aHR] 1.02, 95% CI 0.41–2.56; *p* = 0.969), nor with the requirement for RRT (aOR 1.86, 95% CI 0.50–6.61; *p* = 0.340).

**Table 3:** Association between VExUS ≥ 2 and outcomes.

Outcomes	Unadjusted		Adjusted^a^	
	Effect estimate (95% CI)	*p*-value	Effect estimate (95% CI)	*p*-value
MAKE-30	OR 5.45 (1.69–24.53)	0.010	OR 5.68 (1.59–27.77)	0.014
30-day mortality	HR 1.06 (0.43–2.61)	0.891	HR 1.02 (0.41–2.56)	0.969
Serum creatinine ≥ 200% of baseline	OR 4.71 (1.67–15.50)	0.006	OR 4.25 (1.38–15.05)	0.016
Renal replacement therapy	OR 2.43 (0.84–6.75)	0.092	OR 1.86 (0.50–6.61)	0.340

Effect estimates are presented as odds ratios (ORs) for binary outcomes and as hazard ratios (HRs) for time-to-event outcomes. ORs were estimated using logistic regression and HRs were estimated using Cox proportional hazards models. ^a^Adjusted for APACHE II score, SOFA score, coronary heart disease, heart failure, hypertension, and chronic kidney disease. The reference level was VExUS < 2. CI, confidence interval; MAKE-30, major adverse kidney events at 30 days.

When individual venous ultrasound components were analyzed separately, abnormal Doppler flow patterns of the hepatic, portal, and intrarenal veins were each significantly associated with an increased risk of MAKE-30 (OR 3.62, OR 3.14, and OR 2.42, respectively; all *p* < 0.05), whereas inferior vena cava diameter was not associated with MAKE-30 (Supplementary Additional file 2: Supplementary Table S1).

### 3.4 Survival analysis for MAKE-30

**Figure 3:**
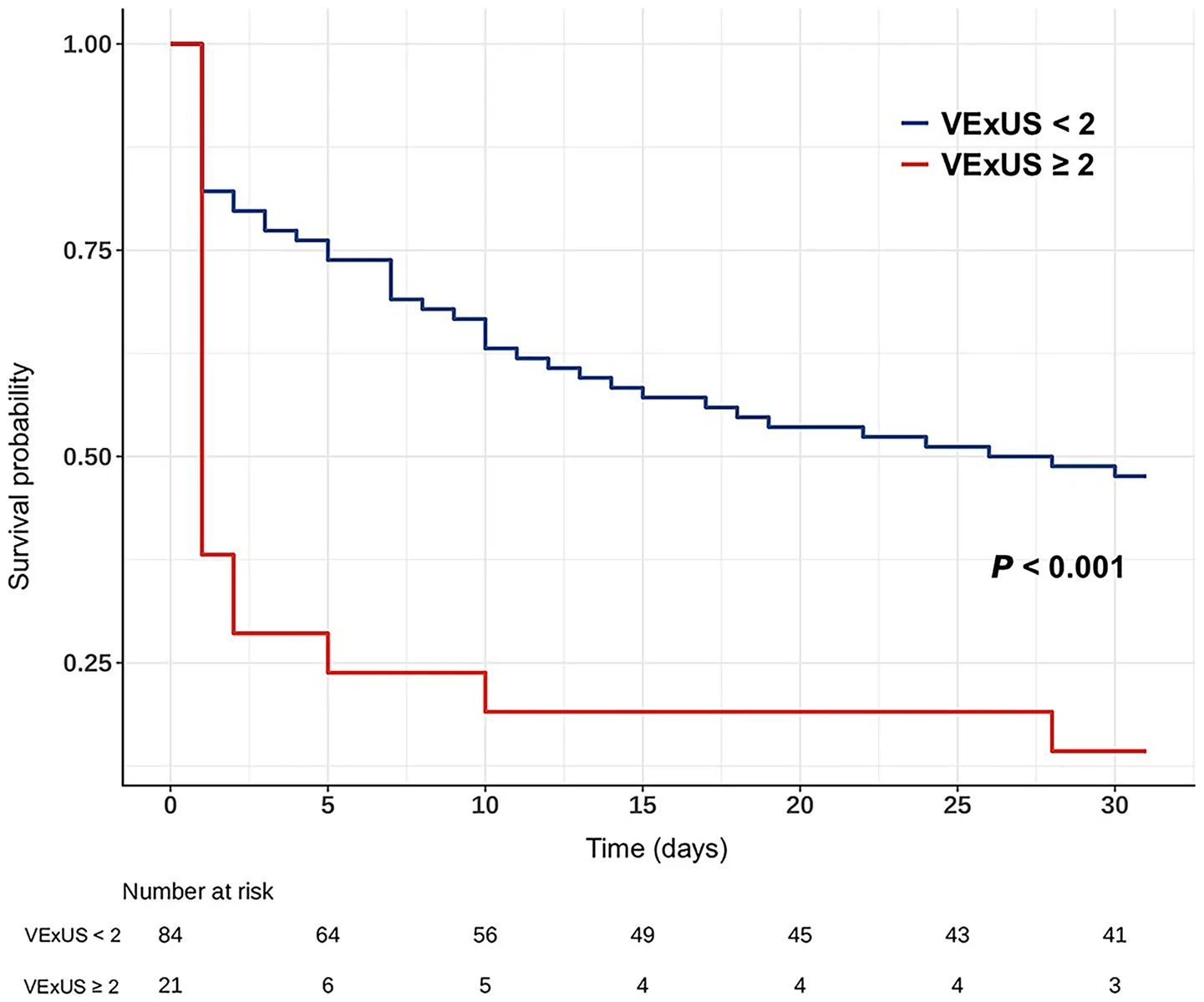
Kaplan–Meier curves for MAKE-30 according to VExUS grade (≥2 vs. < 2). VExUS, Venous excess ultrasound; MAKE-30, Major adverse kidney events at 30 days.

Kaplan–Meier analysis demonstrated a significantly higher cumulative incidence of MAKE-30 among patients with a VExUS grade ≥2 compared with those with a VExUS grade <2 (*p* < 0.001, log-rank test; Figure 3). The survival curves separated early after ICU admission and remained clearly distinct throughout the follow-up period.

## 4 Discussion

In this multicenter prospective cohort study of critically ill elderly patients, we found that 20% of patients exhibited moderate to severe venous congestion (VExUS ≥2) within the first 24 h. After adjustment for illness severity and relevant comorbidities, VExUS grade ≥2 remained associated with MAKE-30. This association was primarily driven by severe AKI (serum creatinine reaching ≥200% of the baseline value), rather than by mortality or the need for RRT. These observations suggest that venous congestion may contribute to organ-specific renal injury rather than merely reflecting global illness severity.

The prevalence of early venous congestion in our cohort is consistent with previous ICU studies (12, 18), indicating that venous congestion is not uncommon even in the early phase of critical illness. This observation may be partly explained by the unique physiological characteristics of the study population. Elderly patients commonly exhibit reduced cardiovascular compliance and diminished renal functional reserve, and frequently have comorbid cardiovascular conditions such as hypertension, CHD, or heart failure (19). These factors may predispose patients to venous congestion even in the presence of mild fluid accumulation or redistribution.

The central finding of our study was that VExUS ≥2 was independently associated with MAKE-30 and with serum creatinine reaching ≥200% of the baseline value. This observation is consistent with previous studies. Klompmaker and colleagues reported a significant association between VExUS and MAKE-30 in a general ICU population (12). Similar associations between VExUS and AKI have also been reported in other clinical settings, including cardiorenal syndrome and acute coronary syndrome (11, 20). VExUS scores correlate with elevations in right atrial pressure (RAP) and have been shown to predict RAP more accurately than inferior vena cava diameter alone, supporting the concept that VExUS reflects systemic venous congestion (21). Elevated RAP increases renal venous pressure, reduces the effective perfusion gradient, and may impair glomerular filtration, contributing to renal dysfunction related to venous congestion (22–24). Abnormal venous Doppler patterns captured by the VExUS system, such as a diastolic-only intrarenal venous flow pattern, directly reflect elevated renal venous pressure and impaired renal venous outflow (25, 26). Importantly, VExUS captures the complex interplay among cardiac function, vascular compliance, and volume status, rather than serving as a direct measure of intravascular volume alone (27). These mechanistic insights are particularly relevant in elderly patients, who often exhibit reduced cardiovascular compliance and preexisting cardiac conditions. Such features may amplify venous congestion and partly explain the observed association. By focusing on critically ill elderly patients, our study underscores the impact of age-related cardiovascular vulnerability on renal outcomes.

However, some studies in ICU populations have not observed a significant association between VExUS and AKI (13, 18, 28). These inconsistent findings may be explained by several factors. First, differences in outcome definitions may contribute to the discrepancy. In the present study, severe kidney injury was defined as serum creatinine reaching ≥200% of the baseline value, thereby focusing on clinically meaningful renal dysfunction. In contrast, some previous studies adopted broader AKI definitions that included patients with Kidney Disease: Improving Global Outcomes (KDIGO) stage 1 AKI. Such definitions may capture transient or minor fluctuations in serum creatinine, which could attenuate the observed association between venous congestion and renal outcomes. Second, the influence of ICU-related interventions should also be considered. Common therapeutic measures in critically ill patients, particularly positive-pressure ventilation, may alter portal and hepatic venous Doppler waveforms, thereby affecting the accuracy of VExUS grading (29). Finally, the heterogeneous etiologies and complex pathophysiology of AKI in ICU populations may obscure the independent contribution of venous congestion. Importantly, the present study extends the prognostic relevance of VExUS to the population of critically ill elderly patients, a group that has been underrepresented in previous studies.

From a clinical perspective, VExUS enables rapid, noninvasive bedside assessment of venous congestion in the ICU, facilitating early identification of moderate to severe congestion and supporting risk stratification. Importantly, it shifts the focus from intravascular volume status alone toward a more integrated evaluation of fluid tolerance. In patients who are fluid-responsive but exhibit significant venous congestion, excessive resuscitation may exacerbate organ congestion, underscoring the need for cautious fluid management. However, the relationship between VExUS findings and specific therapeutic decisions remains incompletely defined, reflecting the complex interplay among volume status, venous compliance, cardiac function, and ongoing interventions (30). Current evidence does not yet support using VExUS to guide fluid therapy. However, higher VExUS grades may indicate reduced diuretic responsiveness, while effective diuretic treatment can improve VExUS scores and restore normal intrarenal venous flow (28, 31). Therefore, VExUS should be considered as part of a multimodal hemodynamic and clinical assessment, integrated with dynamic perfusion markers, echocardiographic findings, and overall clinical trajectory, rather than as a standalone tool to direct therapeutic decisions.

Several limitations of this study should be acknowledged. First, the observational design limits causal inference, and residual confounding cannot be fully excluded despite multivariable adjustment. Detailed data on fluid balance, mechanical ventilation settings, intra-abdominal pressure, and time-varying vasoactive drug doses were not included. These factors may affect venous congestion and renal perfusion, potentially contributing to residual confounding. Second, the relatively small sample size, particularly in the group with venous congestion, may have limited the statistical power for secondary outcomes and contributed to wide confidence intervals. Third, VExUS was assessed at a single early time point after ICU admission; continuous or repeated measurements could provide a more comprehensive understanding of the dynamic evolution of venous congestion and its relationship with outcomes. Fourth, the predominantly elderly cohort, high burden of cardiovascular comorbidities, and extensive exclusion criteria may limit generalizability to broader ICU populations. VExUS assessment is also operator-dependent. Although all stored examinations were independently reviewed and discrepancies resolved by consensus, interobserver agreement was not quantified, limiting assessment of reproducibility and clinical applicability. Future studies should evaluate standardized training and automated image analysis to improve reproducibility. Finally, although VExUS was associated with MAKE-30, we did not collect detailed fluid management data, precluding assessment of whether VExUS-guided interventions could modify clinical outcomes. Future large randomized trials are needed to determine whether VExUS-guided decongestion and dynamic monitoring can improve renal outcomes and support individualized fluid management in critically ill elderly patients.

## 5 Conclusion

In critically ill elderly patients, early venous congestion assessed by the VExUS grading system is not uncommon and independently associated with an increased risk of MAKE-30. This association is primarily driven by severe AKI. These findings support the use of VExUS as a practical bedside tool for identifying elderly ICU patients at high risk of congestion-related renal injury. Further studies are needed to validate whether therapeutic adjustments guided by VExUS can improve renal outcomes in elderly patients.

## Data Availability

The raw data supporting the conclusions of this article will be made available by the authors, without undue reservation.

## Glossary

AKI: Acute kidney injury
ALT: Alanine aminotransferase
APACHE II: Acute Physiology and Chronic Health Evaluation II
APTT: Activated partial thromboplastin time
BNP: Brain natriuretic peptide
CHD: Coronary heart disease
CI: Confidence interval
CKD: Chronic kidney disease
CVP: Central venous pressure
eGFR: Estimated glomerular filtration rate
HR: Hazard ratio
ICU: Intensive care unit
IVC: Inferior vena cava
KDIGO: Kidney Disease: Improving Global Outcomes
MAKE-30: Major adverse kidney events at 30 days
OR: Odds ratio
PI: Portal vein pulsatility index
POCUS: Point-of-care ultrasound
RAP: Right atrial pressure
RRT: Renal replacement therapy
SOFA: Sequential organ failure assessment
VExUS: Venous excess ultrasound
