# Supplementary material for: Association between venous excess ultrasound score and major adverse kidney events at 30 days in critically ill elderly patients: a multicenter prospective cohort study
Source: Front Med (Lausanne). 2026 Aug 6;13:1928971. doi: 10.3389/fmed.2026.1928971 (PMC13490065; doi:10.3389/fmed.2026.1928971)
Supplement: Supplementary file 1 [file Table_1.DOCX]

**1.VExUS Examination Protocol**

The Venous Excess Ultrasound (VExUS) grading system, originally described by Beaubien-Souligny et al., is a structured ultrasonographic approach integrating inferior vena cava (IVC) diameter assessment with Doppler evaluation of the hepatic, portal, and intrarenal veins to quantify the severity of systemic venous congestion.

All ultrasound examinations were performed within 24 hours of ICU admission by trained operators according to a standardized four-step protocol.

**1.1 Inferior Vena Cava (IVC) Assessment**

**Image acquisition:** Patients were examined in the supine position and examination table was adjusted to operator waist height.The probe was placed 1–2 cm inferior to the xiphoid process with the marker oriented toward 3 o’clock to obtain a transverse IVC view using the liver as an acoustic window. The probe was then angled toward the patient’s right side to center the IVC. Afterward, the probe was rotated 90° counterclockwise (from 3 o’clock to 12 o’clock position) to obtain the longitudinal view of the IVC entering the right atrium and the confluence of hepatic veins.

The maximal IVC diameter was measured in B-mode approximately 1–2 cm caudal to the hepatic vein–IVC junction.

**Interpretation criteria:**

IVC diameter < 2 cm: no significant venous congestion (VExUS grade 0); examination may be terminated.

IVC diameter ≥ 2 cm: considered dilated and further Doppler assessment required

**1.2 Hepatic Vein Doppler Assessment**

**Image acquisition:**Subcostal longitudinal view at the hepatic vein–IVC junction.Approximately 3 cm proximal to the IVC confluence during quiet spontaneous breathing.

**Waveform interpretation:**

Normal: S wave ≥ D wave (S ≥ D), with S wave below baseline (toward the heart).

Mild abnormality: S < D, but S remains below baseline.

Severe abnormality: Reversal of S wave above baseline (flow away from the heart).

**1.3 Portal Vein Doppler Assessment**

**Image acquisition:** The portal vein was visualized through an oblique intercostal window. Color Doppler was used to confirm vessel identity (thick hyperechoic walls and hepatopetal flow). Pulsed-wave Doppler sampling was performed centrally within the vessel lumen.

Maximum velocity (Vmax) and minimum velocity (Vmin) were measured to calculate the portal pulsatility index (PI): PI= (Vmax − Vmin)/(Vmax).

**Interpretation criteria：**

Normal: PI < 30% (continuous or minimally pulsatile waveform)

Mild abnormality: PI 30–49%

Severe abnormality: PI ≥ 50%

**1.4 Intrarenal Vein Doppler Assessment**

**I****mage acquisition:**Obtain a coronal or transverse view of the kidney. At the mid‑kidney level, identify interlobar or interlobular veins. Patients were instructed to briefly hold their breath when feasible. Doppler angle was minimized to reduce measurement error. Spectral Doppler frequently demonstrated simultaneous arterial (above baseline) and venous (below baseline) signals.

**Interpretation criteria:**

Normal: Continuous monophasic venous flow

Mild abnormality: Biphasic discontinuous flow (S and D waves)

Severe abnormality: Monophasic discontinuous flow (diastolic phase only)

**二．VExUS Grading Criteria**

Based on the classification proposed by Beaubien-Souligny et al., the present study adopted the C-type VExUS grading system, given prior evidence demonstrating an association between VExUS (type C)≥2. and acute kidney injury.

**Grade 0:**No IVC dilation (IVC < 2 cm), regardless of Doppler findings.

**Grade 1:**IVC ≥ 2 cm with only normal or mildly abnormal patterns.

**Grade 2 (Moderate congestion):**IVC ≥ 2 cm with at least one severe abnormality in at least one Doppler pattern;

**Grade 3 (Severe congestion):**IVC ≥ 2 cm with severe abnormalities in multiple Doppler patterns.
