# Supplementary material for: Association between venous excess ultrasound score and major adverse kidney events at 30 days in critically ill elderly patients: a multicenter prospective cohort study
Source: Front Med (Lausanne). 2026 Aug 6;13:1928971. doi: 10.3389/fmed.2026.1928971 (PMC13490065; doi:10.3389/fmed.2026.1928971)
Supplement: Supplementary file 2 [file Table_2.DOCX]

Table 1 Association of Hepatic, Portal, Renal, and Inferior Vena Cava Doppler Abnormalities with Clinical Outcomes

|  | **OR (95% CI)** | **P-value** |
| --- | --- | --- |
| **MAKE-30** |  |  |
| Hepatic vein | 3.62 (1.32–11.73) | 0.019 |
| Portal vein | 3.14 (1.39–7.42) | 0.007 |
| Renal vein | 2.42 (1.10–5.45) | 0.030 |
| Inferior Vena Cava Diameter | 1.87 (0.85–4.15) | 0.119 |
| **30-day mortality** |  |  |
| Hepatic vein | 0.78 (0.26–2.12) | 0.643 |
| Portal vein | 1.89 (0.80–4.54) | 0.150 |
| Renal vein | 2.11 (0.88–5.29) | 0.099 |
| Inferior Vena Cava Diameter | 0.62 (0.26–1.47) | 0.282 |
| **Serum creatinine ≥200% of baseline** |  |  |
| Hepatic vein | 3.03（1.20-8.19） | 0.022 |
| Portal vein | 2.62（1.20-5.87） | 0.017 |
| Renal vein | 1.80（0.83-3.95） | 0.138 |
| Inferior Vena Cava Diameter | 2.30（1.06-5.11） | 0.038 |
| **RRT requirement** |  |  |
| Hepatic vein | 2.25（0.82-5.99） | 0.106 |
| Portal vein | 1.54（0.62-3.85） | 0.346 |
| Renal vein | 1.21（0.49-3.04） | 0.678 |
| Inferior Vena Cava Diameter | 2.23（0.89-6.02） | 0.096 |
| **AKI** |  |  |
| Hepatic vein | 1.90（0.74-5.36） | 0.199 |
| Portal vein | 1.93（0.87-4.42） | 0.112 |
| Renal vein | 1.75（0.80-3.90） | 0.165 |
| Inferior Vena Cava Diameter | 2.60（1.17-5.93） | 0.020 |

*OR* odds ratio*, CI* confidence interval, *MAKE-30* major adverse kidney events at 30 days, *RRT* renal replacement therapy, *AKI* acute kidney injury

Table 2 Association between VExUS grade and outcomes

|  | **Unadjusted OR (95% CI)** | | | **Adjusted****^a^ OR (95% CI)** | | | |
| --- | --- | --- | --- | --- | --- | --- | --- |
|  | **OR** | **95% CI** | **P-value** |  | **OR** | **95% CI** | **P-value** |
| **MAKE-30** | | | | | | | |
| VExUS 0 | Ref |  |  | Ref | |  |  |
| VExUS 1 | 1.14 | 0.48-2.74 | 0.768 | 0.98 | | 0.37-2.58 | 0.972 |
| VExUS 2 | 3.20 | 0.86-15.58 | 0.105 | 2.28 | | 0.52-12.33 | 0.293 |
| VExUS 3 | 4.08×10^07 | 0.00- | 0.990^b^ | 5.84×10^07 | | 0.00- | 0.988^b^ |
| **30-day mortality** | | | | | | | |
| VExUS 0 | Ref |  |  | Ref | |  |  |
| VExUS 1 | 0.54 | 0.22-1.31 | 0.170 | 0.37 | | 0.14-0.98 | 0.045 |
| VExUS 2 | 0.98 | 0.33-2.92 | 0.968 | 0.81 | | 0.27-2.47 | 0.714 |
| VExUS 3 | 0.66 | 0.15-2.88 | 0.582 | 0.47 | | 0.09-2.34 | 0.354 |
| **Serum creatinine ≥200% of baseline** | | | | | | | |
| VExUS 0 | Ref |  |  | Ref | |  |  |
| VExUS 1 | 1.45 | 0.60-3.53 | 0.409 | 1.28 | | 0.48-3.37 | 0.620 |
| VExUS 2 | 3.88 | 1.10-16.02 | 0.043 | 3.31 | | 0.82-15.31 | 0.102 |
| VExUS 3 | 12.06 | 1.93-235.03 | 0.025 | 10.56 | | 1.31-229.26 | 0.051 |
| **RRT requiremen** | | | | | | | |
| VExUS 0 | Ref |  |  | Ref | |  |  |
| VExUS 1 | 1.77 | 0.61-5.30 | 0.294 | 1.77 | | 0.48-6.83 | 0.395 |
| VExUS 2 | 2.28 | 0.52-9.08 | 0.249 | 1.81 | | 0.32-9.72 | 0.486 |
| VExUS 3 | 5.13 | 1.03-26.24 | 0.043 | 4.01 | | 0.45-37.73 | 0.211 |

*OR* odds ratio*, CI* confidence interval, *MAKE-30* major adverse kidney events at 30 days, *RRT* renal replacement therapy. ^a^Adjusted for APACHE II score, SOFA score, coronary heart disease, heart failure, hypertension, and chronic kidney disease. ^b^ All patients in the Vexus grade 3 group developed MAKE-30, rendering the upper limit of the confidence interval inestimable.
